# Effect of Acetylsalicylic Acid on Biological Properties of Novel Cement Based on Calcium Phosphate Doped with Ions of Strontium, Copper, and Zinc

**DOI:** 10.3390/ijms25147940

**Published:** 2024-07-20

**Authors:** Tamara Vlajić Tovilović, Sanja Petrović, Miloš Lazarević, Aleksandar Pavić, Nikola Plačkić, Aleksa Milovanović, Miloš Milošević, Vesna Miletic, Djordje Veljović, Milena Radunović

**Affiliations:** 1 School of Dental Medicine, University of Belgrade, 11 000 Belgrade, Serbia; tamara.vlajic@stomf.bg.ac.rs (T.V.T.); sanja.petrovic@stomf.bg.ac.rs (S.P.); milos.lazarevic@stomf.bg.ac.rs (M.L.); 2 Institute of Molecular Genetics and Genetic Engineering, University of Belgrade, 11 000 Belgrade, Serbia; sasapavic@imgge.bg.ac.rs (A.P.); nikola.plackic@imgge.bg.ac.rs (N.P.); 3 Faculty of Mechanical Engineering, University of Belgrade, 11 000 Belgrade, Serbia; amilovanovic@mas.bg.ac.rs (A.M.); mmilosevic@mas.bg.ac.rs (M.M.); 4 Faculty of Medicine and Health, Sydney Dental School, University of Sydney, Surry Hills, NSW 2010, Australia; vesna.miletic@sydney.edu.au; 5 Faculty of Technology and Metallurgy, University of Belgrade, 11 000 Belgrade, Serbia

**Keywords:** calcium phosphate, hydroxyapatite, antibiofilm, dental pulp stem cells, zebrafish

## Abstract

This study aimed to compare the biological properties of newly synthesized cements based on calcium phosphate with a commercially used cement, mineral trioxide aggregate (MTA). Strontium (Sr)-, Copper (Cu)-, and Zinc (Zn)-doped hydroxyapatite (miHAp) powder was obtained through hydrothermal synthesis and characterized by scanning electron microscopy (SEM), X-ray diffraction (XRD), and energy dispersive X-ray spectrometry (EDX). Calcium phosphate cement (CPC) was produced by mixing miHAp powder with a 20 wt.% citric acid solution, followed by the assessment of its compressive strength, setting time, and in vitro bioactivity. Acetylsalicylic acid (ASA) was added to the CPC, resulting in CPCA. Biological tests were conducted on CPC, CPCA, and MTA. The biocompatibility of the cement extracts was evaluated in vitro using human dental pulp stem cells (hDPSCs) and in vivo using a zebrafish model. Antibiofilm and antimicrobial effect (quantified by CFUs/mL) were assessed against *Streptococcus mutans* and *Lactobacillus rhamnosus*. None of the tested materials showed toxicity, while CPCA even increased hDPSCs proliferation. CPCA showed a better safety profile than MTA and CPC, and no toxic or immunomodulatory effects on the zebrafish model. CPCA exhibited similar antibiofilm effects against *S. mutans* and *L. rhamnosus* to MTA.

## 1. Introduction

Dental caries is one of the most common diseases impacting the human population worldwide. According to the Global Oral Health Status Report released in 2023 by the World Health Organization (WHO), it is estimated that 2 billion people suffer from caries of permanent teeth and 514 million children suffer from caries of primary teeth globally, with more than two billion cases reported [1]. Recent research has shown that caries is caused by a disruption of the ecological balance within the dental biofilm in favor of acidogenic and aciduric bacteria, primarily *Streptococcus mutans* and *Lactobacillus* species (spp.) [2,3]. *Lactobacillus* spp. does not serve as the incipient factor in dental caries initiation but rather contributes to the subsequent progression thereof. Throughout the developmental stages of dental caries, a synergistic association arises between *Lactobacillus* spp. and *Streptococcus mutans*, facilitating the colonization of *Lactobacillus* strains. These organisms, characterized by their acidogenic and aciduric properties, thrive within the acidic milieu of carious lesions. Consequently, the microbial community within such lesions undergoes a reduction in diversity, thereby fostering the proliferation of acid-tolerant microorganisms, notably *Lactobacillus* species [4]. *Lactobacillus* spp. exhibits greater prevalence in deep caries lesion of cases of symptomatic irreversible pulpitis, compared to normal pulp and reversible pulpitis, which implies that if present the probability of pulp treatment necessity is increased [4]. 

Conventional caries treatment usually includes the complete removal of all demineralized and contaminated dentin, leading to tooth structure loss and weakening of the overall tooth integrity, microbial invasion due to protective barriers disruption, increased risk of pulpitis or pulp necrosis due to pulp exposure, and increased tooth sensitivity, as well as issues with the long-term durability of restorations [5,6,7]. Therefore, this traditional concept was changed and, instead of completely removing carious tissue, the prevention of new and the treatment of existing carious lesions is focused on “arresting” or controlling it, through a minimally invasive treatment approach [8]. Such an approach could be layering caries-affected dentin with restorative materials, limiting the supply of nutrients to bacteria, which further leads to their “starvation” and inactivation of the carious lesion itself. Even though it shows a high success rate, this treatment could fail in a small number of cases. This could be explained by the ability of deep-caries-lesion bacteria to obtain nutrients via pulpal fluids circulating through the dentinal tubules, even if sealed beneath restoration [9]. Therefore, an antimicrobial property of material could be beneficial in eliminating the surviving bacteria.

Success of this kind of minimally invasive approach partially depends on the material which covers the lesion [10]. While calcium hydroxide was considered as the gold standard among the materials for covering pulp and lesions near the pulp tissues, new materials based on hydraulic calcium silicate cement have attracted much attention. Calcium silicate materials are superior to standard calcium hydroxide due to the greater release of calcium ions and consequently increased proliferation and differentiation of dental pulp stem cells [11,12,13]. However, due to the disadvantages of calcium silicate cements such as the inability to completely remineralize dentin, its insufficient adhesion to dentin, and discoloration of the tooth as well as difficult manipulation, there is a necessity for further research in order to develop materials that would meet all requirements [14,15,16].

In recent decades, bioceramic materials based on calcium phosphate have gained significant popularity owing to their notable attributes, including biocompatibility, bioactivity, osteoconductivity, and the similarity of their structure and chemical composition to the inorganic phase of bone and teeth [17,18,19]. Calcium phosphate cements (CPCs) have been extensively studied for various applications, including large bone reconstruction, bone defect filling in cranial and maxillofacial surgery, and as carriers of bone therapeutic substances such as antibiotics, biologically active ions, etc. [20,21,22,23]. Also, in dentistry, CPCs were developed as materials for root canal fillings, and as indirect and direct pulp capping agents where reparative dentin formation occurred in 75% of cases, and the mineral content in caries-affected dentin reached that present in healthy dentin [24,25,26]. They also showed potential in remineralizing enamel, thus acting as a preventative against caries formation [27]. Some studies enriched already used dental materials, such as calcium silicates and adhesives, with hydroxyapatite (HAp) particles, thus showing their ability to enhance biological and mechanical properties [28,29]. However, despite the promise of CPCs as biomimetic dental materials, they show inadequate mechanical properties and lack antibacterial effect [30,31,32,33]. Incorporating citric acid (CA) in the composition of CPC improves its performance in the sense of increasing compressive strength and optimizing setting time. Citrates play a crucial role in the composition of craniofacial bones, teeth, and periodontal tissues, contributing significantly to bone formation and essential bone qualities like stability, strength, and fracture resistance [34]. 

One highly effective and practical approach for promoting cell–material interactions and enhancing antibacterial properties is the introduction of therapeutic ions such as strontium (Sr), copper (Cu), and zinc (Zn) into the hydroxyapatite lattice [35,36,37,38]. Studies have revealed that these ions stimulate the proliferation of human dental pulp stem cells (hDPSCs) and trigger their odontogenic differentiation, while suppressing osteoclast differentiation [39,40,41,42,43].

Dental cement applied to caries-affected dentin should not only serve as a barrier for nutrients but also must possess antimicrobial and antibiofilm properties to combat residual bacteria. Persistent infections are becoming increasingly difficult to treat, prompting a growing interest in repurposing existing drugs, such as nonsteroidal anti-inflammatory drugs (NSAIDs), for biofilm control due to the decreasing effectiveness of currently available antibiotics [44,45]. Acetylsalicylic acid (ASA) is one of the prime candidates for drug repurposing due to its well-documented safety profile in humans [46]. In vitro studies have highlighted the antimicrobial efficacy of ASA demonstrating significant activity against a spectrum of pathogens in suspensions, including *Candida albicans*, *Pseudomonas aeruginosa*, *Escherichia coli*, and *Helicobacter pylori* [47,48]. Additionally, ASA has shown notable antibiofilm effects against *Escherichia coli* [47], *Pseudomonas aeruginosa* [49], *Staphylococcus aureus* [50], and *Candida albicans* [47,51,52].

Even though the above-mentioned components individually somewhat improve the material’s characteristics, combining them could potentially have a synergistic effect. For this reason, the aim of this study was to develop a novel CPC doped with Sr, Cu, and Zn with the addition of citric acid as a potential material for caries-affected dentin layering, and examine its physico-chemical properties, mechanical characteristics (setting time and compressive strength), and in vitro bioactivity. Moreover, the goal was to evaluate the effect of ASA addition on its biological characteristics (biocompatibility in dental pulp stem cells and in zebra fish model, antibiofilm potential against *Streptococcus mutans* (*S. mutans*) and *Lactobacillus rhamnosus* (*L. rhamnosus*) monomicrobial biofilms).

## 2. Results

### 2.1. Characterization of the Sr-, Cu-, and Zn-Doped Hydroxyapatite (Multi-Ion Doped HAp-miHAp)

**Figure 1:**
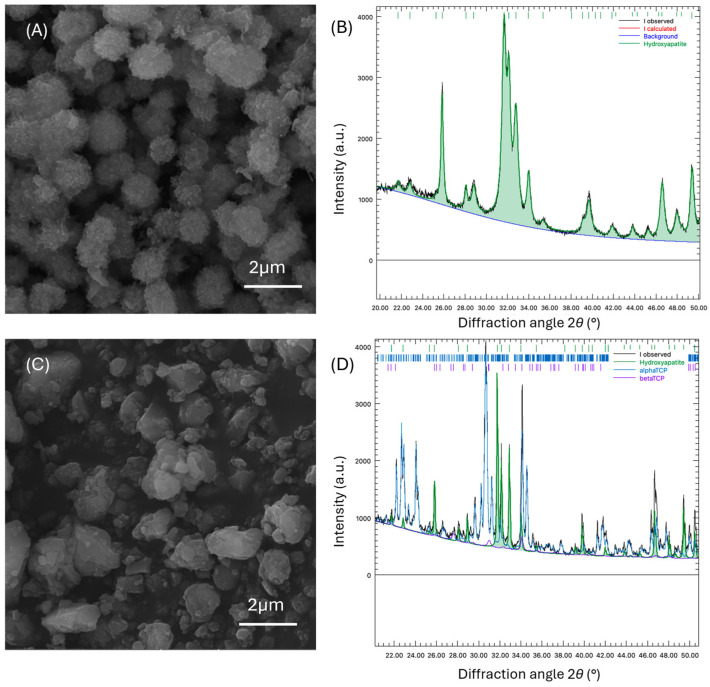
Scanning electron micrographs (SEM) of miHAp powder, (**A**) prior and (**C**) after calcination, and corresponding XRD patterns (**B**,**D**).

Following modified hydrothermal synthesis, the morphology and crystal structure of the multi-ion doped (miHAp) powder was characterized by scanning electron microscopy (SEM), X-ray diffraction (XRD), and energy dispersive spectrophotometry (EDX). SEM images of the miHAp powder obtained prior to calcination revealed the presence of spherical micrometric agglomerates along with nanoscale nanorods (Figure 1A). SEM micrographs of the calcinated and milled powder showed the presence of nanometric and micrometric particles (Figure 1C). XRD on specimens of calcinated powder showed α—tricalcium phosphate (α-TCP) as the dominant phase (Figure 1D). The XRD patterns also confirmed diffraction maximums corresponding to the untransformed HAp phase and revealed peaks associated with a relatively small amount of % β-tricalcium phosphate (β-TCP) (Figure 1D). Representation of HAp phases in percentages was as follows: 27.09 mass% pure HAp, 5.21 mass% β-TCP, and 67.7 mass% α-TCP.

EDX analysis provided data on the atomic percentages of calcium (Ca), phosphate (P), Sr, Cu, and Zn as detailed in Table 1. This analysis identified the presence of Sr and Cu, as well as non-stoichiometric HAp powder, with a Ca/P molar ratio below 1.67. Based on the atomic percentage of all ions, the calculated Ca/P ratio for uncalcinated powder was 1.27, while it was 1.25 for the calcinated powder. The Ca, Sr, Cu, Zn/P ratio for uncalcinated powder was 1.31, while it was 1.36 for the calcinated powder.

### 2.2. Setting Time and Compressive Strength

The setting time of CPC was approximately 5 min (4.83 ± 0.29). The compressive strength was 34.16 ± 4.66 MPa after three days and 23.97 ± 4.39 MPa after 15 days of incubation in simulated body fluid (SBF).

### 2.3. In Vitro Bioactivity

**Figure 2:**
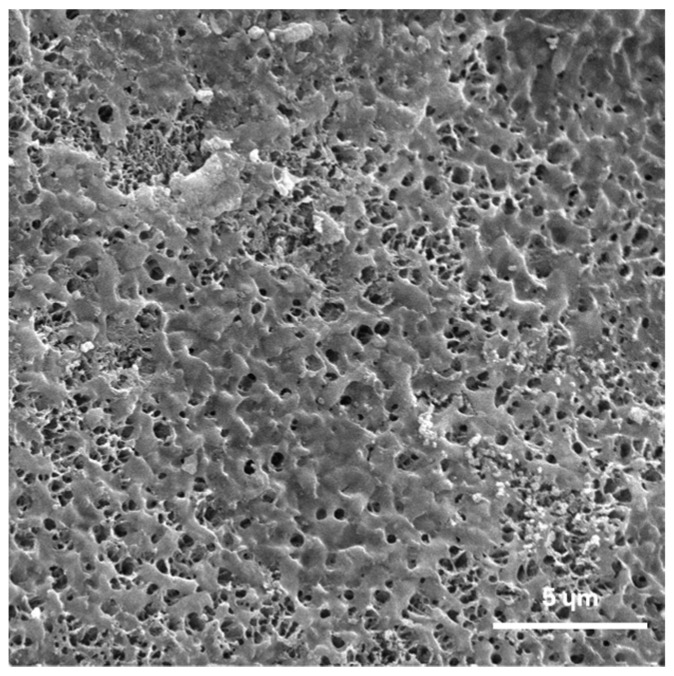
Surface of CPC after 15 days of incubation in SBF, showing the presence of HAp.

In vitro bioactivity refers to the capacity of a material to develop an apatite layer on its surface when immersed in SBF. The results of in vitro bioactivity assessed by SEM after 15 days of incubation in SBF revealed the presence of the HAp layer throughout the surface of the CPC material, as it can be observed in Figure 2.

### 2.4. Viability of Human Dental Pulp Stem Cells (hDPSCs) in the Presence of Cement Extracts

**Figure 3:**
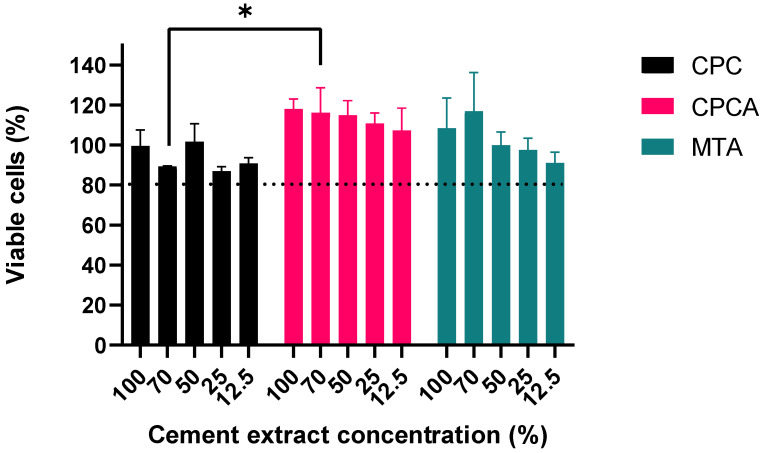
The viability of hDPSC cells at various cement extract concentrations (12.5%, 25%, 50%, 75%, and 100%) assessed by MTT assay; * <0.05 (ANOVA followed by Bonferroni post hoc assay). The dotted line represents 80% of cell viability. Values above 80% indicate that the material is not cytotoxic.

To examine the effect of the new materials (CPC, and CPC with ASA (CPCA)) on hDPSC cells, their viability was evaluated, after 24 h of treatment, by the MTT (3-(4,5-dimethylthiazol-2-yl)-2,5-diphenyltetrazolium bromide) assay (Figure 3) and compared with that of mineral trioxide aggregate (MTA). The data obtained showed that none of the tested materials negatively affected the viability of hDPSC cells (the viability of the cells was above 80%). Moreover, the CPCA extracts stimulated cell proliferation in a dose-dependent manner, resulting in a cell viability rate of 118% at a CPCA extract concentration of 100%. Notably, the viability of hDPSCs cells was higher when treated with CPCA than with CPC, with statistical significance at 70% concentrations (*p* < 0.05, Two-Way ANOVA followed by Bonferroni post hoc assay). Stimulation of cell proliferation (the viability of the cells was above 100%) was also observed in the treatments with 100% and 75% MTA extracts.

### 2.5. In Vivo Toxicity Analyses on Zebrafish

**Figure 4:**
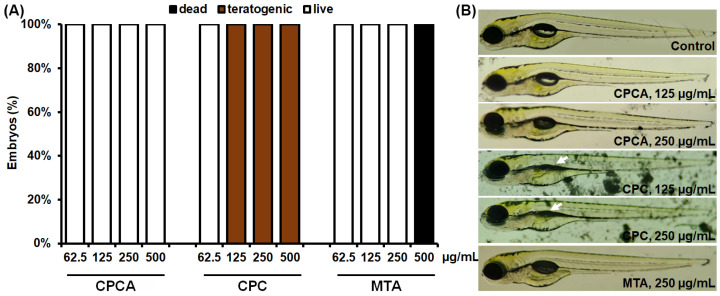
Evaluation of the toxicity of CPCA, CPC, and MTA in the zebrafish model. The effects of the different concentrations of the applied material on (**A**) AB embryos’ survival and teratogenicity and (**B**) morphology are shown. CPC prevented the swim bladder from inflating (arrow).

To investigate the possibility of new dental materials (CPC and CPCA) for human use, their toxicity profile was studied in vivo in zebrafish (*Danio rerio*) and compared with that of MTA, a commercially available and clinically used material. AB zebrafish embryos were exposed to concentrations ranging from 31.3 µg/mL to 500 µg/mL, and systematically and carefully analyzed for the different toxicity endpoints (Figure 4). The in vivo data obtained in this assay showed that CPCA did not induce side effects at the applied concentrations, in contrast to MTA, which was lethal to zebrafish embryos at a dose of 500 µg/mL, and CPC, which impaired swim bladder development (inflation) (Figure 4B). These data indicate that the new CPCA material has a much better safety profile than CPC and MTA and was therefore investigated further in vivo.

**Figure 5:**
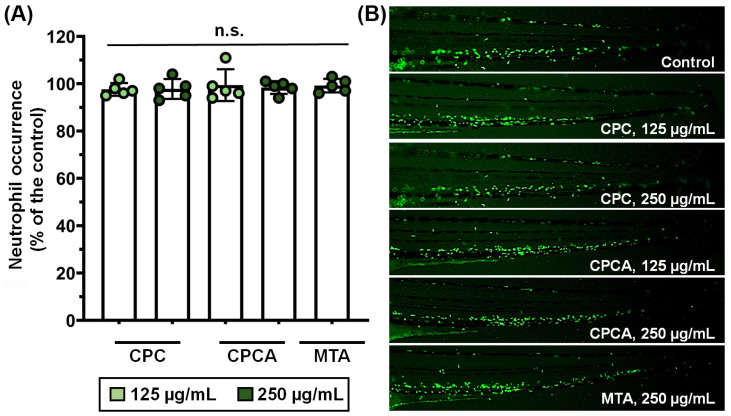
The biocompatibility of dental materials **CPC**, **CPCA**, and **MTA** assessed in the transgenic *Tg* (*mpx*:GFP) i114 zebrafish line with fluorescently labeled neutrophils. (**A**) Neutrophil occurrence and (**B**) fluorescence intensity is shown. No statistically significant difference between the control (untreated) and treated embryos was detected (*p* > 0.5, ANOVA and Bonferroni post hoc assay).

Considering that biocompatibility is one of the critical problems hindering the application of new dental materials, we firstly examined the possible inflammatory or immunosuppressive (neutropenia) effect of CPCA, CPC, and MTA in vivo. Embryos of the reporter zebrafish line *Tg*(*mpx*:GFP) i114, which express green fluorescent protein (GFP) in their neutrophils, were exposed to non-toxic concentrations of these materials of 125 and 250 µg/mL and analyzed for neutrophil occurrence based on their fluorescence (Figure 5). The data obtained in this assay showed that none of the materials used induced an inflammatory response or an immunosuppressive reaction in the treated embryos, as the number of neutrophils was neither increased nor decreased compared to that in the control (untreated) group.

**Figure 6:**
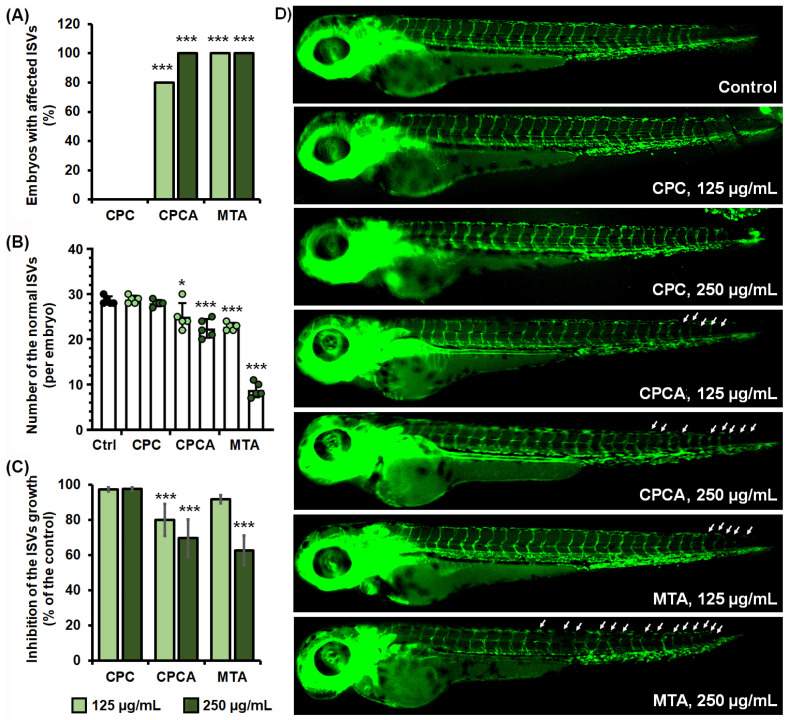
The effect of dental materials CPC, CPCA, and MTA on the intersegmental vessel (ISV) development assessed in the transgenic zebrafish line *Tg*(*fli1*:EGFP) with fluorescently labeled vasculature. The effect on the ISV development was investigated by analyzing (**A**) the frequency of embryos affected in the ISVs (not developed, reduced in size, wrong patterning) (*n* = 10 embryos), (**B**) the number of ISVs affected (n = 5 embryos), and (**C**) the effects on the ISV growth. (**D**) The morphology of the transgenic embryos indicating the affected ISVs (arrow). Statistically significant differences between the treated and untreated groups were determined using ANOVA and Bonferroni test (* *p* < 0.5, *** *p* < 0.001).

Next, to assess the effect of development of the dental material of the new vessels, we examined their effect on the intersegmental vessel (ISV) development in the reporter zebrafish embryos whose endothelial cells express GFP. As can be seen in Figure 6, CPC has no effect on the development of ISVs at the administered doses of 125 and 250 µg/mL, in contrast to MTA, which showed severe adverse effects, especially at the highest administered dose of 250 µg/mL, at which numerous ISVs did not develop or did not develop properly, accompanied by reduced embryo growth (Figure 6B). On the other hand, CPCA showed a slight inhibitory effect on ISV development and no effect on embryo survival at the end of the 5-day treatment.

### 2.6. Antibiofilm Effect

**Figure 7:**
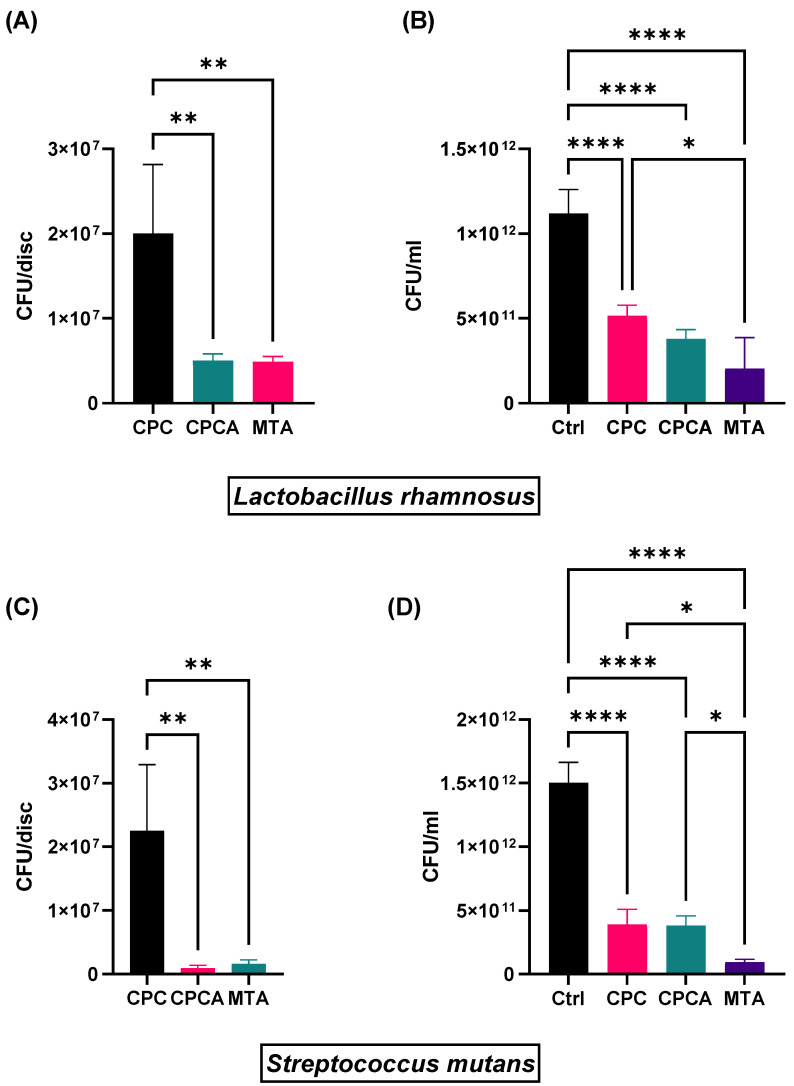
Antibiofilm effect on (**A**) *L. rhamnosus* and (**C**) *S. mutans* on discs, and antimicrobial effect on (**B**) *L. rhamnosus* and (**D**) *S. mutans* in surrounding medium. (One-Way ANOVA followed by Bonferroni test * *p* ≤ 0.05; ** *p* ≤ 0.01; **** *p* ≤ 0.0001).

The numbers of colony forming units (CFUs) obtained from *L. rhamnosus* and *S. mutans* biofilms on discs, and from the medium surrounding discs are shown in Figure 7. ANOVA analysis showed there was a significantly lower number of *L. rhamnosus* CFUs on discs of CPCA and MTA compared to CPC discs (Bonferroni post hoc test, *p* = 0.004; *p* = 0.0038, respectively). No significant difference in CFUs on discs was observed between CPCA and MTA. A similar trend was observed on discs with *S. mutans* biofilms. The number of CFUs was significantly lower on CPCA and MTA discs compared to CPC discs (Bonferroni post hoc test, *p* = 0.0018; *p* = 0.0022, respectively). In the control medium without material, the CFU count was significantly higher, up to approximately 10 times, compared to all test groups for both bacterial species (Bonferroni post hoc test, *p* ≤ 0.0001). 

## 3. Discussion

In this study, a calcium phosphate cement doped with Sr, Cu, and Zn (miHAp) was synthesized. In order to improve its biological properties, ASA was added to the cement’s liquid component. Characterization of miHAp powder, which included SEM, XRD, and EDX, was performed to describe the size and morphology of the powder particles, and dominant phases, and prove the presence of minerals and ions. miHAp powder consisted of relatively uniform rounded agglomerates composed of rod-like HAp nanoparticles. After calcination and milling, the powder was composed of nanometric and micrometric particles of undefined shape, with a dominant α-tricalcium phosphate (α-TCP) phase and with the presence of Sr and Cu. Since Zn was not confirmed, it can be assumed that the zinc content in the powder was below the detection limit of the EDX detector used. Compared to β-TCP, α-TCP has numerous beneficial characteristics, such as solubility in water-based solutions, its ability to undergo hydration at physiological pH levels, and the formation of calcium-deficient hydroxyapatite (CDHA) upon setting [53].

To replicate the properties of calcified tissues, many researchers have developed CPCs by incorporating citric acid. A study showed that the addition of CA (1.5–2 wt.%) to the liquid component of gelatin-modified calcium phosphate cement resulted in increased compressive strength and resistance to washout. This enhancement is thought to be linked to improved paste injectability due to the presence of negatively charged α-TCP particles enveloped by CA molecules [54]. In another study, a CPC formulation with chitosan and CA exhibited favorable outcomes. The inclusion of 20 wt.% and 45 wt.% CA positively impacted compressive strength, reduced setting time, and enhanced the biocompatibility of the experimental cement [55]. 

Compressive strength is another important material characteristic. Several authors have documented increased compressive strength in cement formulations that used CA solutions. This enhancement was explained by its capacity to adhere to calcium phosphates, thereby inhibiting particle agglomeration and facilitating their interaction with the liquid phase. Consequently, this promotes the hydration reaction [54,55,56]. Since the extent of hydration of α-TCP and the precipitation of CDHA crystals are correlated with the compressive strength of cement, it could be suggested that through favoring a hydration reaction CA influenced the mechanical properties. Additionally, Sr doping could also enhance the values of compressive strength, as shown in the literature [57]. Similarly, in our results, a rise in the compressive strength of α-TCP cement samples following a 7-day submersion in phosphate buffered saline (PBS) was noticed. However, extended immersion for 28 days led to a decrease in strength. This decline was associated with the gradual hydrolysis of the cement, leading to increased cement porosity which, over time, can compromise the material’s mechanical property [58]. Compared to the results published by other authors, our results of compressive strength after two weeks (23.97 ± 4.39 MPa) show similar values to those of MTA (23 ± 17 MPa) [59].

Biological properties are important from the aspect of tissue response and the success rate of treatment. However, from the aspect of clinical use, a shorter setting time is crucial. Researchers have suggested different terms and methods for measuring cement hardening time [59,60]. In this study, following the ISO 9917–1 standard (Dentistry—Water-Based Cements—Part 1: Powder/Liquid Acid-Base Cements), for water-based dental materials, the “net setting time” is defined as: “the duration from the end of mixing until the material has set, as determined by specified criteria and conditions.” In order to decrease the long setting time of CPC made with the standard liquid, sodium hydrogen phosphate (exceeding 30 min) [61], we used CA solution as the liquid component. Our results showed that the setting time of CPC (approximately 5 min) was as it is recommended for this type of cement (2.5 to 8 min) by ISO standard 9917-1. It is possible to suggest that the presence of CA decreased the pH value, consequently making the α-TCP powder more soluble. As a result, faster dissolution occurred, facilitating rapid hydration reactions and the formation of a crystalline hydrate network. In a study conducted by Pina et al., it was observed that, when magnesium-substituted calcium phosphate cement was mixed with CA solution at a concentration of up to 15% by weight, it led to faster hydration and a shorter setting time, typically completing in under 5 min [61]. From the literature data, MTA showed a setting time ranging from 41 min to 290 min [59,62,63].

To assess their biocompatibility, the newly synthesized materials with the addition of ASA were tested for cytotoxicity on hDPSCs and a zebrafish model. Notably, CPC alone, as well as with the addition of ASA, did not exhibit any cytotoxic effects on hDPSCs. Furthermore, the addition of ASA induced a higher proliferation of cells in our study, which may be explained by previous findings showing that ASA increases the expression of genes, such as interleukin (IL) 2, 4, and 10, colony stimulating factor (CSF) 3, fibroblast growth factor (FGF) 2, 7, and 9, bone morphogenetic protein (BMP) 2 and 10, and vascular endothelial growth factor (VEGF) C and A, that potentially could activate biological functions related to cell proliferation, regeneration, and differentiation [64,65]. Similar to our study, it has been shown that ASA affected hDPSC proliferation, promoted odontogenic differentiation, leading to mineral nodule formation and increased alkaline phosphatase (ALP) activity [66]; it also stimulated odontogenesis of human dental stem cells and induced the production of transforming growth factor beta 1 (TGF-β1) from dentin [67]. 

Previous studies have shown that biologically active ions can influence cell proliferation and osteogenic differentiation. For example, Sr induced the secretion and growth of dentin sialo phosphoprotein and dentin matrix protein 1 (DSPP, DMP1), crucial molecular triggers in the odontogenic differentiation of hDPSCs, and positively affected the formation of a dentin-like matrix [39]. Furthermore, the introduction of Sr into bioactive materials has been associated with increased osteoblastic activity and simultaneous suppression of osteoclastic differentiation [41,42]. Similarly, bioactive glass enriched with Zn in contact with hDPSC has shown enhanced proliferation and ALP activity, along with increased production and secretion of DSPP and DMP1 [40]. Also, the ability of zinc chloride to induce proliferation of hDPSCs has been reported [43]. Additionally, the combination of Sr and Zn had a synergistic effect, showing that cell viability remained high even at elevated Zn concentrations (up to 6 wt.%). This could indicate that Sr effectively impaired the cytotoxic effect of Zn, enhancing the material’s biocompatibility [68]. However, in our study, doped CPC did not show a cytotoxic effect, but also did not stimulate cell proliferation, which might be due to the relatively small concentrations of Sr and Zn ions in miHAp.

The indication for the potential use of the experimental materials (CPA, CPCA) is the covering of deep carious lesions located near the pulp tissue. Therefore, from the perspective of cell-line selection, the hDPSC model is the closest to in vivo conditions. DPSCs in vitro lack the influence of the immune system, which plays an important role in the host response to dental materials. This absence can lead to an incomplete understanding of biocompatibility and potential inflammatory responses. For that reason, the zebrafish model was utilized. To assess the safety and biocompatibility of new dental materials, animal studies are indispensable. In recent years, the zebrafish model (*Danio rerio*) has proven to be a universal biotechnological platform for the preclinical in vivo safety analysis of bioactive materials and their formulations used in dentistry, including dental materials, tooth-paste compounds, mouth rinses, gels, lozenges, chewing gums, sprays, etc. [69,70,71,72,73,74], and for extrapolation of the data to humans due to their genetic, physiological, and pharmacological similarities [69,70,71,75]. The use of this model system in early preclinical development significantly accelerates entry into clinical trials and increases their success. In addition, this model provides a reliable and ethical alternative to conventional mammalian models and fulfils the 3R principles [76]. 

In this study, we investigated the biocompatibility of new dental materials, CPC and CPCA, compared to the commercially used material, MTA, in a zebrafish model. The optical transparency and the use of different transgenic lines with fluorescently labeled inner organs, such as immune cells, involved in this study provide the unique opportunity to investigate the biosafety of new materials in depth by analyzing real-time effects and the appearance of multiple toxicity endpoints throughout treatment exposure.

Our results showed that CPCA had a better overall safety profile compared to CPC and MTA. At a concentration of 500 µg/mL, MTA was lethal to zebrafish embryos, which is in line with a study of Makkar et al. [70], while CPC significantly impaired swim bladder development, a critical indicator of developmental toxicity. In contrast, CPCA caused no detectable side effects at the concentrations tested, suggesting its potential as a safer alternative for dental applications. The study of Praskova et al. [77] indicated that the zebrafish embryos tolerate relative high level of ASA, with the LC50 value above 200 mg/L. Since the biocompatibility of new dental materials is the critical factor for their safe long-term use, special attention is given to study the zebrafish embryos’ inflammatory response upon CPC and CPCA administration. We used the transgenic zebrafish line *Tg*(*mpx*:GFP)i114, which allowed us to investigate the occurrence of neutrophils as a hallmark of an inflammatory response following exposure to new materials, and showed that none of the materials, namely CPCA, CPC, and MTA, elicited either an inflammatory (neutrophil burden) or immunosuppressive (neutropenia) response. This was shown by the unchanged number of neutrophils in the treated embryos compared to the control group, indicating that these materials did not induce harmful immune responses at the concentrations tested. Further testing of the effect of the materials on angiogenesis in *Tg*(*fli1*:EGFP) zebrafish embryos with fluorescently labeled endothelial cells showed that MTA seriously impeded ISV formation, resulting in significant developmental defects, while CPCA and CPC showed little and no inhibitory effect on ISV development, respectively, further supporting their biocompatibility.

Although selective caries removal has an up to 2.5 times higher success rate than complete caries removal, this minimally invasive technique has shown failure in 12% of cases [6,78,79]. Therefore, one of the most important characteristics for a material used for layering the caries-affected dentin, which might still be contaminated, is an antimicrobial effect. Numerous studies have demonstrated an antimicrobial effect of Cu and Zn against various microorganisms [36,37,68]. Zinc can compete with bacteria for iron uptake, which can compromise the metabolism of bacteria [80], while the main antimicrobial mechanism of copper involves the production of reactive oxygen species (ROS), which cause irreversible damage to the membranes of microorganisms [81]. Also, NSAIDs, including ASA, beside their primary anti-inflammatory effect, have been shown to exhibit antimicrobial properties. NSAIDs are suggested to act as protonophores, thereby diminishing adenosine triphosphate (ATP) production, which is essential for the biochemical processes involved in biofilm formation [82]. Since it is shown that ASA can enhance the effects of antibiotics by influencing the permeability of the bacterial cell, thus allowing antibiotics to reach their targets [83], we can assume that, in the same manner, ASA could allow the penetration of doped ions of Cu and Zn. This could explain the better antibiofilm effect of CPCA compared to CPC. Having in mind that *S. mutans* and *L. rhamnosus* represent the most common bacteria implicated in deep dentin caries development, the new doped materials have been tested for their antimicrobial and antibiofilm activities. Since *S. mutans* is the main etiological factor in caries development, it is the most used species in studies regarding restorative dentistry materials [84]. *Lactobacillus* spp. has been proven in deep carious lesions and is dominant in subjects with symptomatic irreversible pulpitis, a condition that inevitably needs pulp therapy [4]. To the best of our knowledge, there are no studies regarding the antimicrobial or antibiofilm effects of ASA on these microorganisms.

In our study, we compared the CFUs in biofilms grown on the most widely used commercial material, MTA, to CFUs on experimental materials, CPC and CPCA. In *L. rhamnosus* biofilms grown on MTA and CPCA, there is a similar number of CFUs, whereas the amount of CFUs from biofilms grown on CPC was approximately four times greater. In *S. mutans* biofilms, the amount of CFUs from biofilms grown on MTA and CPCA was significantly lower than on CPC (approximately 23- and 13-fold lower, respectively). This undoubtedly shows that the addition of ASA improves the antibiofilm properties of CPC. 

Besides having an antibiofilm effect on its surface, the material should also influence the surrounding environment. In our experiment, we compared bacterial growth in the medium surrounding materials (MTA, CPC, and CPCA) and the medium without material. We confirmed that by adding therapeutic ions and ASA to cements there were significantly less bacteria in the surrounding environment. This is supported by the observed decrease in the number of CFUs not only in the presence of CPCA but also in the presence of CPC. This could serve as indirect proof that active antimicrobial components are released from the cements. Similar to our results, nano-copper oxide demonstrated antimicrobial efficacy in the medium against *Streptococcus mutans*, *Lactobacillus casei*, and *Lactobacillus acidophilus* [85]. Additionally, it was demonstrated that zinc oxide (ZnO) particles exhibited potent antibacterial properties against both *S. mutans* and *Lactobacillus casei* [86].

Maintaining the pulp’s health and vitality, and creating minimally invasive, biologically based treatments are central goals in modern clinical dentistry [87]. Presently, restorative dentistry emphasizes bioactive dental materials. As outlined in a recent FDI policy statement, these materials must demonstrate a clear biological and/or chemical mechanism of action while avoiding significant adverse biological effects [88]. Our newly synthesized cement, based on calcium phosphate doped with ions of Sr, Cu, and Zn with the addition of ASA which has an antibiofilm effect and is biologically safer than commercially used MTA, is in accordance with the main goals of the FDI requirements for dental materials. 

## 4. Materials and Methods

### 4.1. Synthesis of Sr-, Cu-, and Zn-Doped HAp Powder

Sr-, Cu-, and Zn-doped HAp powder (multi-ion doped HAp-miHAp) was obtained by modified hydrothermal synthesis [89]. Briefly, Sr, Cu, and Zn dopants were added in 1.5 dm^3^ of precursor solution containing chemicals listed in Table 2. The concentration of Sr, Cu, and Zn ions in the precursor solution was 1 mol%, 0.4 mol%, and 0.2 mol%, respectively, relative to the quantity of Ca ions. After autoclaving at 160 °C, for 3 h at 8 bar pressure, obtained powder was filtered (Quantitative filter paper 125 mm in diameter, Filter-Lab, Filtros, Barcelona, Spain) and flushed with deionized water and air dried at 105 °C. Subsequently, powder was calcinated in high temperature furnace at 1500 °C for 2 h (Elektron, Banja Koviljaca, Serbia) and crushed and milled in an agate mortar (Kefo, Belgrade, Serbia).

### 4.2. Characterization of the Sr-, Cu-, and Zn-Doped HAp Powder 

The characterization of the miHAp was performed by XRD, EDX, and SEM. The phase composition of miHAp powder before and after calcination was determined by XRD analysis performed on diffractometer (Rigaku Corporation, Tokyo, Japan) in the 2θ angle ranging from 20° to 50°, with a scan rate of 0.02° s^−1^. Phase composition was determined by comparing the experimental XRD patterns with standards compiled by the Joint Committee on Powder Diffraction Standards cards: JCPDS 09–0432, JCPDS 09–0169, and JCPDS 09–0348, for HAP, β-TCP, and α-TCP, respectively. The morphology of the powder before and after calcination was observed with SEM TESCAN MIRA 3 XMU (TESCAN, Brno, Czech Republic). Before SEM analysis, all samples were coated with a gold/palladium alloy using a sputter coater (Polaron SC503, Fisons Instruments, East Grinstead, West Sussex, UK). The elemental composition was examined using EDX detector INCAPentaFETx-3 (Oxford Instruments, Oxford, UK) coupled with a Tescan Vega TS 5130MM (TESCAN, Brno, Czech Republic) scanning electron microscope, both operated at 20 keV. The EDX analysis results are presented as the average arithmetic value from three measurements taken from different surface areas of the sample and expressed in atomic percentages. To obtain cement formulation, the miHAp powder was mixed with liquid component containing deionized water and 20 wt.% citric acid at liquid/powder (L/P) ratio 0.4 mL/g.

### 4.3. Compressive Strength

After mixing, the mixed pastes were placed into cylindrical molds, 12 mm high and 6 mm in diameter. The molded samples were incubated for 24 h in a water bath, to allow cements to initially set, at a temperature of 37 °C and a relative humidity of 100%, after which the samples (n = 5, per group) were removed from the mold and immersed in simulated body fluid for three days and 15 days. The simulated body fluid was changed every third day in the group in which the samples were incubated for 15 days. After the incubation period, the samples were washed with distilled and deionized water and then tested on a universal test machine (100 kN load cell capacity, Shimadzu AGS-X, Shimadzu Corporation, Kyoto, Japan), with a transverse tip speed of 1 mm/min. The maximum stress required to cause fracture of the specimens was noted. The average value of compressive strength in MPa, for each group, was obtained from the results of five samples from each group. The value of compressive strength is expressed in megapascals (MPa).

### 4.4. Setting Time

Determination of setting time was performed in accordance with the modified ISO standard 9917-1. For this experiment an indentor weighing 400 ± 5 g with a flat tip of 1 mm diameter was designed for this purpose. Cement was mixed on a glass plate with a metal spatula and placed in Teflon molds (2 mm deep and 5 mm in diameter). The indentor lowered every 30 s, and indentations made were noted. Setting time was measured from the end of mixing the powder and liquid component until no indentation onto the surface of the cement were observed. Setting time was measured in triplicate. To achieve a high relative humidity, between indentations, the samples were covered with gauze soaked in simulated body fluid.

### 4.5. In Vitro Bioactivity of Cement

To evaluate the formation of an apatite layer on the surface of the CPC after immersion in SBF, indicative of in vitro bioactivity, the following procedures were conducted: after mixing, the cement pastes were molded into cylindrical molds, 12 mm high and 6 mm in diameter, and incubated for 24 h in a water bath, at a temperature of 37 °C and a relative humidity of 100% to allow cement to set, after which the samples were removed from the mold and immersed in simulated body fluid for 15 days. The simulated body fluid was changed every third day. Identification of the formed layer of hydroxyapatite on the surface samples was visualized by SEM after 15 days of incubation.

### 4.6. Acetylsalicylic Acid Addition

During the preparation of cement, in order to avoid uneven distribution of ASA particles, ASA (Pharmacy Kamilica, Belgrade, Serbia) was added in a concentration of 4500 µg/g to the liquid component of cement, which was further mixed with powder. For further experiments, three groups were defined: experimental groups (CPC and CPCA) and commercial dental material (MTA, Master-Dent, Dentonics, Monroe, NC, USA).

### 4.7. Biocompatibility Assessment

#### 4.7.1. In Vitro Cytotoxicity Analysis on Dental Pulp Stem Cells (hDPSCs)

##### Isolation, Cultivation, and Characterization of hDPSCs

**Figure 8:**
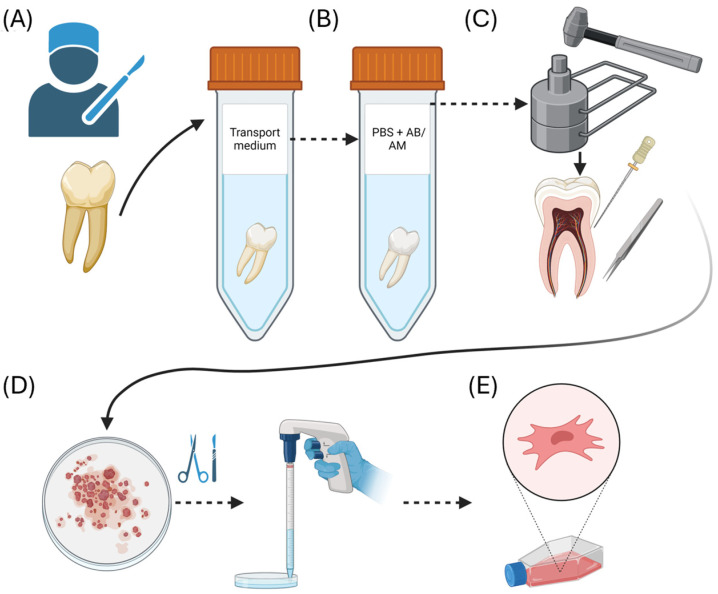
Isolation and cultivation of hDPSC: (**A**) third molar extraction and transportation to the laboratory; (**B**) tooth was cleaned with PBS with antibiotic and antimycotic; (**C**) tissue pulverizer was used for exposing the pulp tissue, which was removed with endodontic instruments; (**D**) dental pulp was cut in small fragments and transported to the T25 flask; (**E**) cultivation of hDPSCs.

hDPSCs were isolated from three semi-impacted wisdom teeth as shown in Figure 8. The donors were healthy patients aged 22–24 years. Teeth were extracted atraumatically, at the Clinic for Oral Surgery, School of Dental Medicine, University of Belgrade, Belgrade, Serbia. Prior to intervention, patients agreed and signed written informed consent. From the Clinic, teeth were transported to laboratory where tooth surfaces were cleaned with PBS (Thermo Fisher Scientific, Waltham, MA, USA). Further hDPSCs were isolated from the samples and processed by following steps: teeth were crushed with sterile tissue pulverizer; dental pulp was extracted using endodontic file; pulp was cut into approximately 1 mm^3^ pieces and transferred to Dulbecco’s Modified Eagle Medium with 10% fetal bovine serum and 1% antibiotic–antimycotic solution (all components are from Thermo Fisher Scientific, Waltham, MA, USA). The samples were incubated at 37 °C, in CO_2_ incubator until cell cultures reached 80% growth confluence, characterized by analyzing the expression of CD90, CD105, CD34, CD73, and CD45 by flow cytometry as previously described [90], and then passaged five times prior to experiment. All procedures were approved by the Ethics Committee of the School of Dental Medicine, University of Belgrade, Serbia (Protocol number 36/2). 

##### Sterilization of Samples

Preceding experiments, components including citric acid and acetylsalicylic acid were UV irradiated for 30 min, distilled deionized water was autoclaved at 160 °C for 2 h, while doped HAp powder was exposed to dry sterilization at 180 °C for 1 h. Sterility of these materials was confirmed. Small samples of dry components were seeded in dextrose broth (Himedia, Mumbai, Maharashtra, India), while distilled deionized water was seeded on blood agar and incubated for 24 h at 37 °C in aerobic conditions after which microbial growth was checked. 

#### Preparation of Cement Extracts 

Samples were extracted in accordance with ISO 10993-12:2009 standard (Biological Evaluation of Medical Devices—Part 12: Sample Preparation and Reference Materials), at mass per extraction volume 0.1 g/mL. Samples were divided into three experimental groups: 1. mineral trioxide aggregate (MTA, Dentonics, Monroe, North Carolina, USA) as the most used commercial material; 2. CPC without ASA; 3. CPCA containing 4500 µg/g of ASA. MTA was prepared according to the manufacturer’s instructions. To obtain 100% extract of experimental cements, each mixed sample of 0.3 g was incubated in 3 mL of complete growth medium containing Dulbecco’s Modified Eagle Medium (DMEM) with 10% fetal bovine serum and 1% antibiotic–antimycotic solution (Thermo Fisher Scientific, Waltham, MA, USA) for 24 h at 37 °C. Incubation was performed in sterile tubes and 100%, 75%, 50%, 25%, and 12.5% test extracts were obtained by diluting 100% extract with complete growth medium. 

##### MTT Assay

Cell viability was assessed in accordance with ISO 10093-5:2009 standard (Biological Evaluation of Medical Devices—Part 5: Tests for In Vitro Cytotoxicity). The experiment was performed in 96 cell culture-treated well plates. hDPSCs were seeded (10,000 cells per well) with 100 µL of complete growth medium and incubated at 37 °C in a humidified 5% CO_2_ environment. After incubation, cells were washed with PBS, exposed to 100 µL of tested extracts (12.5%, 25%, 50%, 75%, and 100%), and incubated for an additional 24 h. Additionally, as control, hDPSCs were incubated only with culture medium. Subsequently, extracts were removed and 100 µL of MTT solution (3---(4,5-dimethylthiazol-2-yl)-2,5-diphenyl tetrazolium bromide) (0.5 mg/mL) (Sigma-Aldrich, St. Louis, MI, USA) was added to each well and incubated for an additional 4 h. After supernatant removal, the formazan crystals were dissolved in 100 µL of DMSO (Sigma-Aldrich, St. Louis, MI, USA) by shaking for 15 min at 37 °C. The optical density (OD) was determined using microplate reader (RT-2100c, Rayto Life and Analytical Sciences Co., Shenzhen, China) at a wavelength of 540 nm. Changes in the metabolic activity, due to the change in cell viability of the sample, correlate with amount of formazan crystal formed. The experiment was performed in triplicate. 

#### 4.7.2. In Vivo Biocompatibility Assessment 

##### Cement Extract Preparation

**Figure 9:**
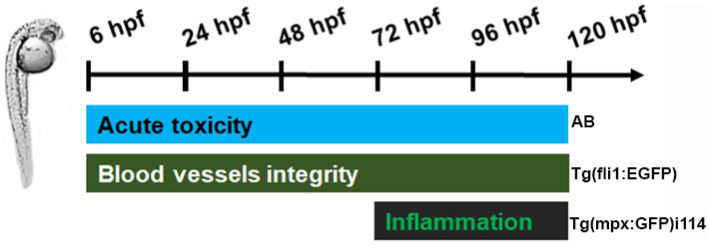
A graphic representation of the in vivo experiments performed in the various zebrafish lines in the different time frames.

The extracts of the tested materials are prepared according to the ISO 10993-12 standard. In brief, 0.2 g of crushed (ground) dental material was dissolved in 1 mL of E3 medium (5 mM NaCl, 0.17 mM KCl, 0.33 mM CaCl_2_, and 0.33 mM MgSO_4_ in distilled water) and incubated for 3 days at 37 °C with shaking at 180 rpm. After this extraction phase, the resulting material suspension was centrifuged at 13,000× *g* for 15 min, and the obtained supernatant was carefully separated from the pellet and immediately used for the following in vivo experiments in the zebrafish (*Danio rerio*) embryo model. A graphic representation of in vivo experiments on the zebrafish embryos is shown in Figure 9.

##### In Vivo Experiments in the Zebrafish Model

All experiments with zebrafish embryos were performed in accordance with the European Directive 2010/63/EU and the Ethical Guidelines for the Care and Use of Laboratory Animals of the Institute of Molecular Genetics and Genetic Engineering of the University of Belgrade. Wild-type (AB) zebrafish embryos, kindly provided by Dr. Ana Cvejić (Wellcome Trust Sanger Institute, Cambridge, UK), were reared to the adult stage in a temperature- and light-controlled zebrafish facility at 28 °C and a standard 14:10 h light–dark photoperiod. The fish were fed twice daily with commercial dry food (SDS200 and SDS300 granular food; Special Diet Services, Essex; UK and TetraMin^TM^ flakes; Tetra Melle, Germany) and daily with *Artemia nauplii*.

##### In Vivo Toxicity Assessment

The toxicity of the dental materials (CPC, CPCA, and MTA) was evaluated in accordance with the general rules of the OECD guidelines for the testing of chemicals [91]. Embryos generated by pairwise mating were washed to remove debris and distributed 10 per well in 24 well plates containing 1 mL of E3 medium, and maintained at 28 °C. To evaluate acute (lethal) and developmental (teratogenic) toxicity, embryos were exposed to the five different concentrations (31.3, 62.5, 125, 250, and 500 µg/mL) of each tested material at 6 h post-fertilization (hpf), an early embryonic stage that ensures high sensitivity to the molecules tested. Treated embryos were examined every day under a stereomicroscope (Carl Zeiss™ Stemi 508 doc Stereomicroscope, Oberkochen, Germany) for the appearance of apical endpoints (Table S1) until 120 hpf. Dead embryos were collected and discarded every 24 h. E3 medium was used as a negative control. Experiment was performed in duplicate with 10 embryos for each concentration. At 120 hpf, embryos were anesthetized by the addition of 0.1% (*w*/*v*) Tricaine solution (Sigma-Aldrich, St. Louis, MO, USA), photographed, and killed by freezing at −20 °C for ≥24 h.

##### Inflammatory and Immunosuppressive In Vivo Response Determination

To assess whether the tested materials have possible inflammatory and/or immunosuppressive effects, 6-hpf old embryos of transgenic *Tg*(*mpx*:GFP)i114 zebrafish line expressing GFP in neutrophils [92] were exposed to the non-toxic concentration (125 and 250 µg/mL) of the tested material, and incubated at 28 °C. At 72 hpf, embryos were imaged under a fluorescence microscope (Olympus BX51, Applied Imaging Corp., San Jose, CA, USA) and the neutrophil occurrence (fluorescence intensity) was determined using ImageJ software. Ten embryos per concentration were used, while five embryos were randomly selected, imaged, and then analyzed for neutrophil occurrence using ImageJ software (version 1.52, NIH public domain software; NIH—National Institutes of Health). The effect of applied treatments was analyzed in relation to the control (untreated) sample.

##### Anti-Angiogenic Potential Evaluation in the Zebrafish Model

The materials’ effect on blood vessel development was investigated in transgenic zebrafish *Tg*(*fli1:*EGFP)^y^ embryos with GFP-expressing endothelial cells [93], as was previously described [94]. Briefly, zebrafish embryos at 6 hpf were exposed to the non-toxic concentration (125 and 250 µg/mL) of the tested material, and incubated at 28 °C. At 72 hpf stage, embryos were anesthetized with 0.02% tricaine and subsequently imaged under a fluorescence microscope (Olympus BX51, Applied Imaging Corp., San Jose, CA, USA) and examined for the development of intersegmental blood vessels (ISVs). Ten embryos per concentration were used. To determine the effect of applied treatment on ISV development, the five embryos were randomly selected and imaged, and the length of ISVs was measured by ImageJ software (version 1.52, NIH public domain software; NIH—National Institutes of Health). The effect of applied treatments was analyzed in relation to the control (untreated) sample.

### 4.8. Antibiofilm Assay

#### 4.8.1. Bacterial Strains and Growth Conditions

Reference strains *S. mutans* ATCC 25175, and *L. rhamnosus* ATCC 53103 (Microbiologics KWIK-STIK, Manassas, VA, USA) were used in the study. *S. mutans* and *L. rhamnosus* were activated by seeding in Brain Heart Infusion (BHI) broth (HiMedia, Mumbai, Maharashtra, India) and De Man, Rogosa and Sharpe (MRS) broth (Becton, Dickinson and Company, Franklin Lakes, NJ, USA), respectively, and incubated at 37 °C for 24 h in microaerophilic conditions in Anaerojar (GasPak, Oxoid, Hampshire, UK). Bacterial suspensions were seeded on blood agar (HiMedia, Mumbai, Maharashtra, India) (*S. mutans*) and MRS agar (Becton, Dickinson and Company, Franklin Lakes, NJ, USA) (*L. rhamnosus*) and incubated at 37 °C for 24 h in the same microaerophilic conditions. After reference strain activation, a few colonies of each bacterial species were transferred to their respective broth. The obtained bacterial suspension was centrifuged at 3000 rpm for 10 min (Lace 6, Colo Lab Experts, Ljubljana, Slovenia); the supernatant formed after centrifugation was removed and the pellet resuspended in sterile PBS and set to turbidity of 0.5 McFarland standard (DEN-1 densitometer, Biosan, Riga, Latvia), which is equivalent to ≈10^8^ bacterial cells/mL. Suspensions were serially diluted with appropriate media to set final concentration to 10^5^ cells/mL. 

#### 4.8.2. Biofilm Formation

Bacterial biofilms were grown on cement discs (n = 4) made of three types of material: MTA, CPC, and CPCA. Discs were made by placing freshly mixed cements into sterile aluminum molds (2 mm depth, 5 mm diameter) and incubated for 24 h at 37 °C, following their removal. A sample disc from each group was checked for sterility as mentioned in the section “Sterilization of Samples”. Monomicrobial biofilms of both bacterial species were formed as follows: discs were covered by 100 µL of sterile artificial saliva for four hours to allow the formation of salivary pellicle after which saliva was removed and 200 µL of bacterial suspension prepared in previous section was poured in each well of 96-well plate. Further, incubation was carried out in static conditions at 37 °C, 48 h in anaerobic (*S. mutans*) or 48 h in microaerophilic conditions (*L. rhamnosus*).

#### 4.8.3. Biofilm CFU Quantification

In order to detach firmly adhered bacterial cells from the discs, discs with formed biofilm were gently rinsed with PBS, transferred to tubes filled with 1 mL of PBS and vortexed for 15 min. Subsequently, 20 µL from each tube was serially diluted in eight tenfold dilutions in PBS and each dilution was seeded onto the agar plate. After incubation (37 °C, 48 h in anaerobic (*S. mutans*) or 48 h in microaerophilic conditions (*L. rhamnosus*)) formed colonies were counted.

#### 4.8.4. Determination of CFUs in Medium Surrounding the Discs

To determine whether antibacterial substances are released from the cements into the environment, the number of CFU/mL in the medium surrounding the discs with bacterial biofilm (after 24 h of incubation) was determined for both bacterial species. A total volume of 20 µL of the medium was collected and 12 tenfold serial dilutions in PBS of each medium were seeded on blood agar (*S. mutans*) and MRS agar (*L. rhamnosus*). After 48 h incubation at 37 °C in microaerophilic conditions, colonies were counted.

### 4.9. Statistical Analysis 

Statistical analyses were performed by the software package GraphPad Prism (ver. 9 GraphPad Software, Inc. San Diego, California, USA). The normality of the distribution was tested using the Kolmogorov–Smirnov test. Afterward, the data were analyzed using the One-Way or Two-Way analysis of variance (ANOVA) test with Bonferroni post hoc test or Kruskal–Wallis test, depending on the distribution of the data. The level of significance was defined as alpha = 0.05.

## 5. Conclusions

The newly synthesized cement based on calcium phosphate doped with strontium, copper, and zinc ions, and with addition of acetylsalicylic acid (ASA), demonstrated antibiofilm properties against monomicrobial biofilms of the cariogenic pathogens *Streptococcus mutans* and *Lactobacillus rhamnosus*, comparable to those of the commercially available dental material, mineral trioxide aggregate (MTA). While none of the tested materials (CPC, CPCA, or MTA) exhibited cytotoxic effects on human dental pulp stem cells (hDPSCs), in vivo testing using a zebrafish model—a universally recognized biotechnological platform for assessing the toxicity of bioactive agents—revealed that CPCA possesses lower toxicity and a better safety profile than MTA. According to these results, future studies should focus on the long-term effects and functional performance of CPCA in dental applications to confirm its suitability for clinical use.

## Figures and Tables

**Table 1:** EDX elemental analysis of miHAp powder. Atomic percentages of each element in both uncalcinated and calcinated powder.

Element	Un-Calcinated Powder (Atomic %)	Calcinated Powder (at. %)
**Ca**	15.30 ± 1.99	15.400 ± 0.74
**P**	12.07 ± 1.90	12.250 ± 0.43
**Sr**	0.54 ± 0.06	0.530 ± 0.06
**Cu**	0.07 ± 0.04	0.075 ± 0.03
**Zn**	0	0

**Table 2:** The formulation of precursor solutions for doped HAp synthesis. * Fisher Scientific (Loughborough, Leicestershire, United Kingdom), ** Kemika (Zagreb, Chroatia), *** E. Merck (Darmstadt, Germany).

Components of Precursor Solution	Mass of Components (g)
Ca (NO_3_)_2_·H_2_O *	10.5800
NaH_2_EDTA·2H_2_O *	11.1800
NaH_2_PO_4_·2H_2_O *	4.6800
Urea *	12.000
Sr (NO_3_)_2_ **	0.0960
Cu (NO_3_)_2_·3H_2_O ***	0.0439
Zn (NO_3_)_2_·6H_2_O **	0.0270

## Data Availability

The raw data supporting the conclusions of this article will be made available by the authors on request.

## Supplementary Materials

The following supporting information can be downloaded at https://www.mdpi.com/article/10.3390/ijms25147940/s1.
