# Supplementary material for: Effect of Acetylsalicylic Acid on Biological Properties of Novel Cement Based on Calcium Phosphate Doped with Ions of Strontium, Copper, and Zinc
Source: Int J Mol Sci. 2024 Jul 20;25(14):7940. doi: 10.3390/ijms25147940 (PMC11276672; doi:10.3390/ijms25147940)
Supplement: Supplementary file 1 [file ijms-25-07940-s001.zip › ijms-3075665-supplementary.pdf]

Table S1. Lethal and teratogenic effects observed in zebrafish (*Danio rerio*) embryos at different hours post fertilization (hpf).

Category	Toxicological parameters	Exposure time (hpf)				
		24	48	72	96	120
**Lethal effect**	Coagulated eggs[a]	●	●	●	●	●
	Lack of the heart beating	●	●	●	●	●
	Non-detachment of the tail	●	●	●	●	●
	Lack of somite formation	●	●	●	●	●
**Teratogenic effect**	Malformation of head	●	●	●	●	●
	Malformation of eyes[b]	●	●	●	●	●
	Malformation of sacculi/otoliths[c]	●	●	●	●	●
	Malformation of chorda[d]	●	●	●	●	●
	Malformation of tail[e]	●	●	●	●	●
	Scoliosis/lordosis	●	●	●	●	●
	Yolk edema[f]	●	●	●	●	●
	Growth retardation[g]		●	●	●	●
	Hatching[h]			●	●	●
	Swimmbladder development[i]					●
**Hepatotoxicity**	Yolk absorption[j]			●	●	●
	Liver darkening[k]			●	●	●
**Cardiotoxicity**	Pericardial edema[l]		●	●	●	●
	Heart beating rate (beat/min) [m]					●

[a] No clear organs structure is recognized
[b] Malformation of eyes was recorded for the retardation in eye development and abnormality in shape and size.
[c] Presence of none, one or more than two otoliths per sacculus, as well as reduction and enlargement of otic vesicles
[d] The abnormality in notochord shape
[e] Tail malformation was recorded when the tail was bent, twisted or shorter than to control embryos as assessed by optical comparison
[f] Enlargement of the yolk sac
[g] Growth retardation was recorded by comparing with the control embryos in a body length (after hatching)
[h] Embryos hatching in a period from 72 hpf to 120 hpf stage
[i] The presence, reduced size or absence of swim bladder
[j] The resorption of yolk
[k] The change in liver color and dark color appearance
[l] An appearance of pericardial sac enlargement
[m] The number of beats within 30 sec
